# Resveratrol Attenuates Heat Stress-Induced Impairment of Meat Quality in Broilers by Regulating the Nrf2 Signaling Pathway

**DOI:** 10.3390/ani12151889

**Published:** 2022-07-25

**Authors:** Yiyang Zhao, Zhen Li, Xiaocheng Wang, Fei Zhao, Chi Wang, Qingyue Zhang, Xingyong Chen, Zhaoyu Geng, Cheng Zhang

**Affiliations:** Department of Animal Science, College of Animal Science and Technology, Anhui Agricultural University, Hefei 230036, China; zyy15856815971@163.com (Y.Z.); li1819559237@163.com (Z.L.); wxc964295363@163.com (X.W.); zhaofei550516@163.com (F.Z.); nicksix6@126.com (C.W.); zqy15205603820@163.com (Q.Z.); chenxingyong@ahau.edu.cn (X.C.); gzy@ahau.edu.cn (Z.G.)

**Keywords:** antioxidant, heat stress, resveratrol, meat quality, signaling pathway

## Abstract

**Simple Summary:**

Broilers easily experience heat stress (HS), especially in summer, due to a lack of sweat glands. HS has unfavorable effects on the production efficiency, meat quality, antioxidant capacity, and welfare of broilers, which can be mitigated with nutritional strategies. Resveratrol (RES) has been found to mitigate the HS-induced decrease of antioxidant capacity in muscle and, therefore, improve the meat quality of broilers under HS. Nevertheless, there are few reports on the mechanism of action of RES on meat quality and antioxidant status in the muscle of broilers subjected to HS. Our study demonstrated that RES could improve the antioxidant ability of muscle in broilers under HS by activating the Nrf2 signaling pathway and ultimately relieving the HS-induced deterioration of meat quality.

**Abstract:**

Studies have indicated that dietary resveratrol (RES) improves the meat quality of broilers subjected to heat stress (HS), but the mechanism of action remains unclear. Therefore, the main purpose of this study was to investigate the effect of RES on meat quality, muscle antioxidant status, and its mechanism of action in broilers under HS. A total of 162 male AA broilers at 21 days old with similar weight were randomly assigned to 3 treatment groups with 6 replicates each. The control group (ambient temperature: 22 ± 1 °C) and HS group (ambient temperature: 33 ± 1 °C for 10 h a day from 8:00 to 18:00 and 22 ± 1 °C for the remaining time) were fed a basal diet and the HS + RES group was fed a basal diet with 400 mg/kg RES. The feeding was conducted for 21 continuous days. The results indicated that HS decreased final body weight (BW), average daily gain (ADG), average daily feed intake (ADFI), breast and leg muscle yield, a*_24h_, pH_24h_, the activities of catalase (CAT), glutathione S-transferase (GST) and glutathione peroxidase (GSH-Px), and mRNA levels of nuclear factor erythroid 2–related factor 2 (*Nrf2*), heme oxygenase-1 (*HO-1*), NADPH quinone oxidoreductase 1 (*NQO1*), and *GSH-Px* (*p* < 0.05). HS also increased b*_45min_, L*_24h_, drip loss, malondialdehyde (MDA) content, and kelch-like epichlorohydrin-associated protein 1 (*Keap1*) mRNA level (*p* < 0.05). Compared with the HS group, the HS + RES group exhibited a higher ADG, breast and leg muscle yield, a*_24h_, pH_24h_, activities of GST and GSH-Px, and mRNA levels of *Nrf2*, *HO-1,* and *NQO1* but had lower drip loss and Keap1 mRNA level (*p* < 0.05). RES can improve meat quality and the muscle antioxidant ability of heat-stressed broilers by activating the Nrf2 signaling pathway.

## 1. Introduction

Heat stress (HS) is a serious problem in global animal production, restricting the development of the livestock and poultry industries. Broilers easily experience HS due to a lack of sweat glands, especially in tropical and subtropical regions [1]. HS increases the breathing rate and heart rate of broilers, obstructs homeostasis, and interferes with the functions of the endocrine and immune systems [2,3,4], which leads to a decrease in growth and reproduction capacity, reducing production efficiency and causing huge economic losses to the broiler industry [5].

Due to higher living standards, more people around the world are seeking high-quality meat and poultry products. HS during poultry production not only directly affects growth performance and carcass traits but also reduces the quality of meat, which becomes pale, soft, and exudative [6]. Previous studies have indicated that HS negatively affects meat color, pH, drip loss, and shear force to varying degrees [7]. Furthermore, when chickens are exposed to continuous high temperatures, reactive oxygen species (ROS) are excessively produced and accumulated in the body, which destroys the balance of redox levels, causing oxidative damage to lipids, proteins, and DNA [8]. Studies have revealed that HS decreases the activities of a variety of antioxidant enzymes in the spleen and serum of heat-stressed broilers [9,10,11], suggesting that HS can cause a decline in antioxidant capacity. As a major transcription factor, nuclear factor E2-related factor 2 (Nrf2) regulates the levels of a series of genes related to antioxidation and protects the body from oxidative stress damage. Our previous study revealed that the low level of *Nrf2* expression in the spleen of broilers under HS led to the restriction of the expression levels of antioxidative factors, including glutathione peroxidase (*GSH-Px*), superoxide dismutase 1 (*SOD1*), and heme oxygenase-1 (*HO-1*) [11]. Given the considerable negative effects of HS on broiler production, finding new nutritional mitigation strategies is urgent.

Resveratrol (RES) is a polyphenol substance that is produced by many plants when they are stimulated, and it acts as an antitoxin [12]. It has various physiological and biochemical effects, including anti-inflammatory, antioxidant, anti-proliferative, and immune regulatory effects [13]. Therefore, it has received research attention in recent years [14]. Previous research in our laboratory found that RES can improve meat quality and the oxidation resistance of heat-stressed broilers; however, the mechanism is still unclear [15]. Moreover, our research confirmed that RES could activate the Nrf2 signaling pathway in the spleen [11] and intestinal tract [16] of broilers under HS and alleviate the oxidative damage caused by HS. A recent study in ducks revealed that RES could alleviate lipopolysaccharide-induced liver peroxidation through the enhanced expression of *Nrf2* and *HO-1* [17]. According to the above findings, we hypothesized that RES possibly improves the muscle antioxidant capacity of broilers subjected to HS through Nrf2 signaling pathway activation in muscle tissue, which improves meat quality. Therefore, this study investigated the effects of RES on growth performance, carcass traits, meat quality, and the muscle antioxidation mechanism of heat-stressed broilers.

## 2. Materials and Methods

### 2.1. Animals, Diets, and Experimental Design

The experimental procedures and animal handling standards were approved by the Anhui Agricultural University Animal Ethics Committee (approval code: 2021189). In the experiment, 300 1-day-old male Arbor Acres (AA) broilers were raised and immunized under routine conditions to the age of 21 days. Subsequently, 162 healthy broilers weighing 875 ± 5 g were selected and randomly distributed into 3 treatment groups with 6 replicates each; each replicate contained 9 birds. The ambient temperature of the control group (CON) was 22 ± 1 °C, and the room temperature of the HS group (HS) and HS + RES group (HS + RES) was controlled at 33 ± 1 °C from 8:00 to 18:00 each day and 22 ± 1 °C for the remaining time. The lighting and relative humidity were set according to the specifications in our previous research [16]. The birds of the CON and HS groups were fed a basal diet, and the birds of the HS + RES groups were fed a basal diet with 400 mg/kg of RES (purity > 98%). The basal diet was prepared as stated in our previous published study [16]. Its composition and nutrient levels are presented in Table S1. The experimental treatment was provided for 21 days. Feed and water were provided ad libitum, and feed consumption was recorded daily.

### 2.2. Carcass Characteristics and Sample Collection

After 21 days of feeding trials, the broilers were weighed after 12 h of fasting, and 2 broilers per replicate were selected for sampling. Slaughter percentage, semi-eviscerated carcass yield, eviscerated carcass yield, breast muscle yield, and leg muscle yield were measured and calculated, as previously mentioned [18]. The right pectoralis major muscle sample was stripped, quickly frozen in liquid nitrogen, and then stored at –80 °C for further analyses. The left pectoralis major muscle was stripped and stored at 4 °C for meat quality analysis.

### 2.3. Meat Quality

The pH of the muscle sample and meat color (L*: lightness, a*: redness, and b*: yellowness) were measured in triplicate at 45 min and 24 h postmortem by using a portable pH meter (PH-STAR, MATTHAUS, Germany) and a colorimeter (CR-400, Minolta Camera, Osaka, Japan), respectively. Approximately 15 g of the muscle sample was cut, weighed, and hung in an inflatable sealing bag for 24 h at 4 °C. Subsequently, the sample was removed, the surface was dried with a filter paper, and reweighed to calculate the drip loss. Muscle samples of approximately 25 g were placed into a sealed bag, heated in a water bath at 80 °C until the inside temperature reached 70 °C, and then cooled to 4 °C. The surface water was removed, and the samples were weighed to calculate the cooking loss. The cooked muscle samples were trimmed along the direction of the muscle fibers and cut into rectangular strips of 2 × 1× 1 cm. Subsequently, the shear force of the samples was measured in triplicate using a muscle tenderness meter (C-LM4-23-68910, Tenderization Instruments, Harbin, China).

### 2.4. Antioxidant Activity and Metabolite Content

A muscle sample (approximately 500 mg) was homogenized in 5 mL of ice-cooled normal saline and centrifuged at 3000× *g* for 10 min at 4 °C. The supernatant was preserved for analysis. The activities of total superoxide dismutase (T-SOD), catalase (CAT), glutathione S-transferase (GST), glutathione peroxidase (GSH-Px), glutathione reductase (GR), and the contents of malondialdehyde (MDA) and protein carbonyl (PC) were determined using commercially available kits (Nanjing Jiancheng Bioengineering Institute, Nanjing, China) following the manufacturer’s instructions.

### 2.5. Real-Time Polymerase Chain Reaction

Total RNA was isolated from the muscle sample (100 mg) using TRIzol TM Reagent (Thermo Fisher Scientific, Waltham, MA, USA), and the concentration and purity were measured according to the ratio of A260/A280 using an Epoch Microplate Spectrophotometer (BioTek Instruments, Inc., Winooski, VT, USA). Two µL of total RNA were reversely transcribed into cDNA using the Hifair^®^ 1st Strand cDNA Synthesis SuperMix for qPCR (gDNA digester plus) kit (Yeasen, Shanghai, China). The Hieff^®^ qPCR SYBR Green Master Mix (Low Rox Plus) kit (Yeasen, Shanghai, China) and Real-Time PCR System (Thermo Fisher Scientific, MA, USA) were used to quantify the mRNA levels of genes. The primers for *Nrf2*, kelch-like epichlorohydrin-associated protein 1 (*Keap1*), *HO-1*, NAD(P)H/quinone oxidoreductase 1 (*NQO1*), *CAT*, *SOD1*, *GST*, *GSH-Px*, and *β-actin* were synthesized by SangonBiotechCo., Ltd. (Shanghai, China) and are presented in Table S2, as in our previous study [16]. The reaction condition and parameter setting were according to the settings in our previous research [19]. The target genes’ relative mRNA expression was determined using the 2^−∆∆Ct^ method against *β-actin* as the housekeeping gene.

### 2.6. Statistical Analysis

The general linear model was applied (Yij = µ + di + εij; Yij: the observation, µ: the general mean, di: the treatment effect, εij: the random error). All data were analyzed by one-way ANOVA in SPSS 18.0 statistical software (SPSS, Inc., Chicago, IL, USA), followed by Duncan’s multiple range tests. Each replicate (*n* = 6) served as the experimental unit for growth performance, and the individual bird (*n* = 12) served as the experimental unit for other indicators, including carcass trait, meat quality, antioxidative enzyme activities, metabolite content, and antioxidant-related gene levels. A level of *p* < 0.05 was considered statistically significant.

## 3. Results

### 3.1. Growth Performance

As shown in Table 1, the final body weight (BW), average daily gain (ADG), and average daily feed intake (ADFI) of the HS group were lower than those of the CON group (*p* < 0.05). However, the negative effects of HS on the ADG were greatly alleviated by RES addition (*p* < 0.05).

### 3.2. Carcass Trait

As displayed in Table 2, birds that experienced HS exhibited a decline in breast and leg muscle yield compared with the control birds (*p* < 0.05). Nevertheless, the negative effects of HS on these yields were alleviated by RES supplementation (*p* < 0.05).

### 3.3. Meat Quality

As presented in Table 3, broilers under HS exhibited a lower b*_45min_, L*_24h_, and drip loss versus heat-unstressed broilers, whereas pH_24h_ and a*_24h_ were significantly reduced (*p* < 0.05). However, the pH_24h_ and a*_24h_ in the HS + RES group were considerably improved compared with the HS group, and drip loss declined significantly (*p* < 0.05).

### 3.4. Antioxidative Enzyme Activities and Metabolite Content

As presented in Table 4, HS decreased CAT, GST, and GSH-Px activities (*p* < 0.05) and increased MDA content compared with the CON group (*p* < 0.05). Additionally, the HS + RES group exhibited higher GST and GSH-Px activities than the HS group (*p* < 0.05).

### 3.5. Antioxidant-Related Gene Levels

Compared with the CON group, the mRNA levels of *Nrf2*, *HO-1*, *NQO1,* and *GSH-Px* (Table 5) in the muscle of broilers under HS exhibited a significant decline (*p* < 0.05), whereas the mRNA level of *Keap1* significantly increased (*p* < 0.05). Compared with the HS group, the mRNA levels of *Nrf2*, *HO-1*, and *NQO1* in the muscle of broilers in the HS + RES group significantly increased (*p* < 0.05), whereas the mRNA level of *Keap1* significantly decreased (*p* < 0.05).

## 4. Discussion

The lack of sweat glands and the increased metabolic heat production make broilers vulnerable to HS [4]. The present study indicated that heat-stressed broilers exhibited lower final BW, ADG, and ADFI, which agrees with previous findings [4,9]. The possible reason is that birds tend to keep themselves cool at high ambient temperature by reducing their feed intake to minimize the production of metabolic heat [20]. Furthermore, HS can cause metabolic disorders and protein breakdown, and additional energy is used to resist oxidative damage caused by HS [21]. Considerable attention has been paid to nutritional strategies for alleviating HS. Previous studies have revealed that RES can improve the growth performance of broilers under HS [9]. Similarly, our present research confirmed that RES improved the growth performance of broilers under HS.

HS led to changes in the carcass traits of broilers, namely a decline in breast muscle rate and an increase in the proportion of abdominal fat [22]. Our previous study found that HS decreased the eviscerated carcass yield [19]. Consistent with these previous findings, the present study indicated that the breast muscle rate, leg muscle rate, and semi-evisceration yield of broilers were significantly decreased by HS, which might be attributed to the decrease in feed intake and the increase in muscle degradation [23]. Previous studies have reported that the plant polyphenols lycopene [24] and epigallocatechin gallate [19] can mitigate the adverse effects of HS on the carcass traits of quail and broilers, respectively. Yu et al. [25] revealed that RES could reduce abdominal fat deposition and improve carcass traits of ducks. Our present study found that RES had a positive effect on the carcass traits of heat-stressed broilers. One possible reason is that RES can improve intestinal development and health, thereby promoting the absorption of protein in the intestine [16].

Meat quality is a comprehensive trait that involves a series of evaluation indicators. Meat color is ordinarily evaluated by L*, a*, and b*, which are determined by the level of myoglobin, hemoglobin, and cytochrome in the muscle. After slaughter, glycolysis causes the accumulation of lactic acid in muscle tissue which decreases the pH value, results in protein denaturation, and decreases the muscle protein’s ability to bind to water [26]. Therefore, the changes in muscle pH value directly affect the water retention ability and tenderness of meat [27]. Previous studies have revealed that HS has negative effects on the meat quality of broilers, such as lower ultimate pH and higher L* and drip loss [7,27,28,29]. Mancini and Hunt [30] reported that HS led to reduced feed intake, which led to the inadequate supply of myoglobin and other proteins that regulate meat color, resulting in an increase in muscle lightness and a decrease in redness. Consistent with these results, the present study found that b*_45min_, L*_24h_, drip loss, and cooking loss were significantly increased by HS, whereas a*_24h_ and pH_24h_ were significantly decreased. Jang et al. [31] suggested that dietary RES could be used as a chicken meat quality enhancer. This study confirmed that RES significantly alleviated the deterioration of meat quality caused by HS, which is consistent with the results of Zhang et al. [7].

High ambient temperature induces the production of ROS, which ultimately results in oxidative damage [32]. Many studies have reported that HS has a negative impact on the antioxidant capacity, reduces the activities of a series of antioxidant enzymes, and thus results in oxidative damage [33,34,35]. In this study, HS caused an obvious decline in the activities of CAT, GSH-Px, and GST in the muscle of broilers and an increase in MDA content. Muscle antioxidant function is positively correlated with meat quality; this is one of the reasons that HS results in decreased meat quality [7,15]. RES, a natural plant polyphenol, has been proven to exhibit outstanding antioxidant function. Yulug et al. [36] reported that adding RES to the diet of mice could reduce MDA content and increase the activities of SOD and CAT. Liu et al. [9] found that adding RES to the diet of black-bone chickens under HS could increase SOD, GSH-Px, and CAT activity and reduce MDA content in serum. Similarly, the present results indicated that dietary RES could significantly increase GST and GSH-Px activities in the muscle of broilers under HS. This was presumed to be a key reason why RES could improve meat quality. The antioxidant effect of RES is closely related to its ability to scavenge free radicals (biological efficiency: about 95%) [37]; high free radical content destroys the antioxidant system and causes tissue damage [38].

In general, Nrf2, as a transcription factor, binds to its inhibitor Keap1 in the cytoplasm in an inactive state and is degraded through the ubiquitin–proteasome pathway; therefore, it exhibits low activity under normal physiological conditions [39]. Under stress conditions, the dissociation of the Keap1–Nrf2 complex results in the stabilization of Nrf2, which is then transferred into the nucleus and stimulates the expression of cellular protective genes, including antioxidant factors such as *HO-1*, *NQO1*, *SOD*, *GST,* and *GSH-Px* [40]. Previous studies have reported that the Nrf2 signaling pathway may be a key to controlling HS-induced oxidative stress [41]. This study revealed that the expression levels of antioxidant-related genes (*Nrf2*, *HO-1*, *NQO1*, and *GSH-Px*) in the Nrf2 signaling pathway in muscle were significantly decreased by HS, whereas the mRNA expression of *Keap1* was significantly increased. This implied that HS inhibited the Nrf2 signaling pathway, which may be the underlying mechanism for the HS-induced decrease in the antioxidant capacity of muscle, ultimately resulting in a decrease in meat quality. Yang et al. [42] reported that RES increased neuronal viability and inhibited neuronal apoptosis by enhancing the Nrf2 signaling pathway activation. Zhang et al. [11] revealed that the mRNA expression levels of *CAT*, *GR*, *MnSOD*, *HO-1,* and *Nrf2* in the spleen of broilers under HS were increased by dietary RES, indicating that the Nrf2 signaling pathway in the spleen might be activated. Similarly, Wang et al. [17] reported that *Nrf2* and *SOD* mRNA levels in the jejunum of heat-stressed boilers could be improved by RES, which may alleviate the inhibitory effect of HS on the Nrf2 signaling pathway. The present study found that *Nrf2*, *HO-1*, and *NQO1* mRNA in the muscle of heat-stressed broilers were remarkably improved by RES. Moreover, our results revealed that RES significantly improved total Nrf2, HO-1, and NQO1 protein levels in muscle and Nrf2 protein levels in the muscle nuclei of broilers subjected to HS (data not published). Therefore, our results suggest that RES not only up-regulated the expression of *Nrf2* in the muscle of broilers under HS but also promoted its translocation into the nucleus, thus promoting the expression of its downstream antioxidation-related genes. This may be the underlying mechanism why RES can improve the muscle antioxidant capacity of heat-stressed boilers and ultimately mitigate the deterioration of meat quality caused by HS.

## 5. Conclusions

HS leads to muscle oxidative stress in broilers, which adversely affects slaughter performance and meat quality. RES could improve the muscle antioxidant ability by activating the Nrf2 signaling pathway in the muscle of heat-stressed broilers and ultimately relieve the deterioration of meat quality caused by HS.

## Figures and Tables

**Table 1:** Effects of RES on the growth performance of heat-stressed broilers.

Items	CON	HS	HS + RES	SEM	*p*-Value
Initial BW (g)	876.95	879.08	878.44	1.154	0.739
Final BW (g)	2334.50 ^a^	2190.17 ^b^	2268.21 ^ab^	24.263	0.029
ADG (g)	69.36 ^a^	62.43 ^b^	66.13 ^a^	1.161	0.026
ADFI (g)	128.10 ^a^	123.07 ^b^	126.88 ^b^	0.794	0.006
F/G	1.85	1.97	1.92	0.024	0.109

(1): BW, body weight; ADFI, average daily feed intake; ADG, average daily gain; F/G, feed to gain ratio. (2): CON, control group; HS, heat stress group; HS + RES, heat stress + resveratrol group. ^a,b^ Values without common superscripts in the same row differ significantly (*p* < 0.05).

**Table 2:** Effects of RES on the carcass traits of heat-stressed broilers.

Items	CON	HS	HS + RES	SEM	*p*-Value
Slaughter percentage (%)	93.00	92.66	93.05	0.085	0.169
Semi-eviscerated carcass yield (%)	88.38	84.71	87.19	0.707	0.093
Eviscerated carcass yield (%)	75.56	74.29	74.79	0.395	0.383
Breast muscle yield (%)	21.38 ^a^	19.87 ^b^	21.06 ^a^	0.231	0.013
Leg muscle yield (%)	16.35 ^a^	14.67 ^b^	15.66 ^a^	0.236	0.001

CON, control group; HS, heat stress group; HS + RES, heat stress + resveratrol group. ^a,b^ Values without common superscripts in the same row differ significantly (*p* < 0.05).

**Table 3:** Effects of RES on the meat quality of heat-stressed broilers.

Items	CON	HS	HS + RES	SEM	*p*-Value
pH_45min_	6.45	6.46	6.44	0.041	0.985
pH_24h_	6.38 ^a^	5.97 ^b^	6.35 ^a^	0.059	<0.001
L*_45min_	49.34	50.46	50.46	0.354	0.325
a*_45min_	10.04	9.20	9.91	0.205	0.227
b*_45min_	13.19 ^b^	15.23 ^a^	14.35 ^ab^	0.324	0.012
L*_24h_	53.44 ^b^	55.65 ^a^	55.26 ^a^	0.372	0.014
a*_24h_	10.44 ^a^	9.34 ^b^	10.30 ^a^	0.210	0.039
b*_24h_	12.84	13.93	13.55	0.252	0.147
Drip loss (%)	1.94 ^b^	2.43 ^a^	2.02 ^b^	0.084	0.012
Cooking loss (%)	22.81	25.66	23.94	0.547	0.065
Shear force (N)	30.55	35.30	32.68	0.964	0.120

(1): L*, lightness; a*: redness; b*: yellowness. (2): CON, control group; HS, heat stress group; HS + RES, heat stress + resveratrol group. ^a,b^ Values without common superscripts in the same row differ significantly (*p* < 0.05).

**Table 4:** Effects of RES on antioxidant status and metabolite content in the skeletal muscle of heat-stressed broilers.

Items	CON	HS	HS + RES	SEM	*p*-Value
T-SOD (U/mg protein)	5.42	5.04	5.27	0.213	0.772
CAT (U/mg protein)	105.90 ^a^	87.45 ^b^	99.25 ^ab^	3.415	0.048
GST (U/mg protein)	469.20 ^a^	382.30 ^b^	439.30 ^a^	14.080	0.006
GSH-Px (U/mg protein)	239.30 ^a^	193.80 ^b^	234.90 ^a^	7.822	0.013
GR (U/mg protein)	1.56	1.45	1.49	0.058	0.783
MDA (nmol/mg protein)	0.39 ^b^	0.64 ^a^	0.53 ^ab^	0.048	0.170
PC (nmol/mg protein)	1.85	2.13	1.90	0.089	0.446

(1): T-SOD, total superoxide dismutase; CAT, catalase; GST, glutathione S-transferase; GSH-Px, glutathione peroxidase; GR, glutathione reductase; MDA, malondialdehyde; PC, protein carbonyl. (2): CON, control group; HS, heat stress group; HS + RES, heat stress + resveratrol group. ^a,b^ Values without common superscripts in the same row differ significantly (*p* < 0.05).

**Table 5:** Effects of RES on antioxidant-related gene levels in the skeletal muscle of heat-stressed broilers.

Items	CON	HS	HS + RES	SEM	*p*-Value
*Nrf2*	1.00 ^a^	0.66 ^b^	0.93 ^a^	0.056	0.001
*Keap1*	1.00 ^c^	1.56 ^a^	1.24 ^b^	0.087	0.002
*HO-1*	1.00 ^a^	0.59 ^b^	0.89 ^a^	0.060	0.001
*NQO1*	1.00 ^a^	0.64 ^c^	0.82 ^b^	0.055	0.002
*CAT*	1.00	0.83	0.88	0.042	0.276
*SOD1*	1.00	0.92	0.96	0.023	0.398
*GST*	1.00	0.90	0.96	0.022	0.181
*GSH-Px*	1.00 ^a^	0.59 ^b^	0.79 ^ab^	0.068	0.012

(1): *Nrf2*, nuclear factor erythroid 2–related factor 2; *Keap1*, kelch-like epichlorohydrin-associated protein 1; *HO-1*, heme oxygenase-1; *NQO1*, NADPH quinone oxidoreductase 1; *CAT*, catalase; *SOD1*, superoxide dismutase 1; *GST*, glutathione S-transferase; *GSH-Px,* glutathione peroxidase. (2): CON, control group; HS, heat stress group; HS + RES, heat stress + resveratrol group. ^a,b,c^ Values without common superscripts in the same row differ significantly (*p* < 0.05).

## Data Availability

The data presented in this study are available on request from the corresponding author.

## Supplementary Materials

The following supporting information can be downloaded at: https://www.mdpi.com/article/10.3390/ani12151889/s1, Table S1: Composition and nutrient levels of the basal diet; Table S2: Primers used for Real-Time PCR.
