# Supplementary material for: Resveratrol Attenuates Heat Stress-Induced Impairment of Meat Quality in Broilers by Regulating the Nrf2 Signaling Pathway
Source: Animals (Basel). 2022 Jul 25;12(15):1889. doi: 10.3390/ani12151889 (PMC9330235; doi:10.3390/ani12151889)
Supplement: Supplementary file 1 [file animals-12-01889-s001.zip › Supplemental Material.pdf]

Table S1. Composition and nutrient levels of the basal diets.

Ingredients (%)		Calculated Nutrient levels	
Corn	57.30	Metabolizable energy, Kcal/kg	3044
Soybean meal	35.20	Crude protein, %	20.02
Soybean oil	3.99	Methionine, %	0.40
Salt	0.30	Ca, %	0.90
$CaHPO_4·2H_2O$	1.61	Available phosphorus, %	0.40
Limestone	1.12	Lysine, %	1.07
DL-Methionine	0.10	Methionine + Cysteine, %	0.73
Choline chloride	0.15		
Premix	0.23		
Total	100.00		

The premix provided the following per kg of diet: vitamin A 4000 IU; vitamin D_3, 800 IU; vitamin E, 44 IU; vitamin K_3, 0.5 mg; thiamine, 1 mg; riboflavin, 3.75 mg; vitamin B_6, 1 mg; vitamin B_{12}, 15 μg; niacin, 10 mg; biotin, 0.2 mg; pantothenic acid, 12 mg; folic acid, 1.3 mg; Cu, 10mg as $CuSO_4·5H_2O$; Fe, 80 mg as $FeSO_4$; I, 0.6 mg as KI; Zn, 100 mg as $ZnSO_4$; Mn, 25 mg as $MnSO_4$; Se, 0.15 mg as Na_2SeO_3.

Table S2. Primers used for Real-Time PCR.

Gene	GenBank ID	Primer sequences (5' to 3')	Length (bp)
β-actin	NM_205518.1	F: TGATATTGCTGCGCTCGTTG	127
		R: AACCATCACACCCTGATGTCTG	
*Nrf*2	NM_205117.1	F: TTCGCAGAGCACAGATACTTC	188
		R: TGGGTGGCTGAGTTTGATTAG	
*Keap*1	XM_025145847.1	F: CTGCTGGAGTTCGCCTACAC	96
		R: CACGCTGTCGATCTGGTACA	
HO-1	NM_205344.1	F: TGTCCCTCCACGAGTTCAAG	181
		R: CTCCAGTTGCTGCCATAGAA	
*NQO*1	NM_001277619.1	F: CTCCGAGTGCTTTGTCTACGA	150
		R: ATGGCTGGCATCTCAAACC	
CAT	NM_001031215.2	F: GGCGTATGACCCTAGCAACA	167
		R: TCTGATAATTGGCCACGCGA	
*SOD*1	NM_205064.1	F: GGAGTGGCAGAAGTAGAAATAGAAG	150
		R: AGGTCCAGCATTTCCAGTTAG	
GST	XM_015284825.2	F: GGAAGCCATTTTAATGACAGA	76
		R: TCCTTTAAAAGCCTGTAGCAGA	
GSH-Px	NM_001277853.2	F: ACGGCGCATCTTCCAAAG	73
		R: TGTTCCCCCAACCATTTCTC	

Nrf2: nuclear factor erythroid 2-related factor 2; *Keap1*: kelch-like epichlorohydrin-associated protein 1; *HO-1*: heme oxygenase 1; *NQO1*: NAD(P)H/ quinone oxidoreductase 1; *CAT*: catalase; *SOD1*: superoxide dismutase 1; *GST*: glutathione S-transferase; *GSH-Px*: glutathione peroxidase.
